# Species Identification of Bovine, Ovine and Porcine Type 1 Collagen; Comparing Peptide Mass Fingerprinting and LC-Based Proteomics Methods

**DOI:** 10.3390/ijms17040445

**Published:** 2016-03-24

**Authors:** Mike Buckley

**Affiliations:** Manchester Institute of Biotechnology, the University of Manchester, Manchester, M1 7DN, UK; m.buckley@manchester.ac.uk; Tel./Fax: +44-161-306-5175

**Keywords:** bone collagen, collagen function, hydroxylation, variability, peptide mass fingerprinting

## Abstract

Collagen is one of the most ubiquitous proteins in the animal kingdom and the dominant protein in extracellular tissues such as bone, skin and other connective tissues in which it acts primarily as a supporting scaffold. It has been widely investigated scientifically, not only as a biomedical material for regenerative medicine, but also for its role as a food source for both humans and livestock. Due to the long-term stability of collagen, as well as its abundance in bone, it has been proposed as a source of biomarkers for species identification not only for heat- and pressure-rendered animal feed but also in ancient archaeological and palaeontological specimens, typically carried out by peptide mass fingerprinting (PMF) as well as in-depth liquid chromatography (LC)-based tandem mass spectrometric methods. Through the analysis of the three most common domesticates species, cow, sheep, and pig, this research investigates the advantages of each approach over the other, investigating sites of sequence variation with known functional properties of the collagen molecule. Results indicate that the previously identified species biomarkers through PMF analysis are not among the most variable type 1 collagen peptides present in these tissues, the latter of which can be detected by LC-based methods. However, it is clear that the highly repetitive sequence motif of collagen throughout the molecule, combined with the variability of the sites and relative abundance levels of hydroxylation, can result in high scoring false positive peptide matches using these LC-based methods. Additionally, the greater alpha 2(I) chain sequence variation, in comparison to the alpha 1(I) chain, did not appear to be specific to any particular functional properties, implying that intra-chain functional constraints on sequence variation are not as great as inter-chain constraints. However, although some of the most variable peptides were only observed in LC-based methods, until the range of publicly available collagen sequences improves, the simplicity of the PMF approach and suitable range of peptide sequence variation observed makes it the ideal method for initial taxonomic identification prior to further analysis by LC-based methods only when required.

## 1. Introduction

For decades, collagen has been considered an important biomolecule with biomedical uses, such as the base of a scaffold for tissue regeneration [1,2], as well as in the food industry in its denatured form of gelatine [3,4]. As the most abundant protein in the extracellular tissues used in animal feed [5], it was also widely used as a cheap source of protein for livestock feed made from a range of animal species that not only included livestock but occasionally fallen exotic animals from zoological gardens and country parks. Following the outbreak of variant Creutzfeldt–Jakob disease, believed caused by the consumption of food contaminated with prions such as cattle tissues of individuals that suffered from bovine spongiform encephalopathy, and the subsequent change in the regulations over the use of processed animal by-products during the 1990s [6], the ability to discriminate the species of the tissues going into the feed became a global concern [7,8]. It is also the most abundant protein to survive into the archaeological and palaeontological records, which can be informative of early forms of animal husbandry [9] as well as environmental changes that occurred during the past [10].

### 1.1. Collagen Structure

Collagen refers to a group of proteins known for its triple helical structure established over 60 years ago [11,12]. There are currently at least 28 types of collagen known across a range of human tissues, some made up of three identical (alpha; α) chains (homotrimers) such as in cartilage (type II collagen), whereas others are made up of genetically distinct chains (heterotrimers), such as the most abundant type in skin and bone, type I collagen, which in mammals contains two identical alpha chains (“α1(I)”) and one genetically distinct chain (“α2(I)”). In order to form the triple helix there is an unusually large relative amount of the imino acids proline and its modified form hydroxyproline, which create the twisting of the helix and play important roles in the stability of the structure [13]. Having the smallest side group of all amino acids to fit within this twisting structure, glycine is present as nearly one in every three amino acids throughout the length of the helical domains. Hydroxylation of lysine residues also occurs in order to form glycosylated residues, mostly with disaccharides, which influences intermolecular cross-linking and fibril formation [14]. This structure makes extracellular tissues particularly stable, not only *in vivo*, but also against decay processes such as heating conditions [5] and pressure-treatments that occur in the animal tissue rendering process, as well as long-term survival in the burial environments in ancient remains [15]. In bone, collagen survival is thought to be enhanced due to the confinement of the triple helix within its mineral bioapatite [16].

### 1.2. Species Identification

New methods of species identification continue to be developed for a range of applications, the most pressing of those being for food products that have important economic implications and an impact on human health [17]. Where food fraud relates to the substitution of one species with another similar but cheaper one unknown to the consumer [18], it may also be important to ensure the absence of some biomolecules (e.g., milk proteins, lactose or gluten) for health reasons. Due to the extent of processing, these examples rely on molecular techniques for species determination.

In the animal feed industry, the components of the feed material retains some level of morphology, by which some taxonomic information can be determined through microscopic analyses, albeit this information has been often limited to separating terrestrial from non-terrestrial species, rather than the desired ability to separate ruminants from non-ruminants [19]. In order to reach these taxonomic levels of discrimination, developments in a range of biomolecular methods have increased the options available for policing future relaxations in the relevant legislations [19,20,21,22,23].

There are also applications to the study of archaeological faunal material, where the species identifications of skeletal remains are used to make inferences about past human ecology and the development of agriculture and human-environment interactions. These rely on interpretations from ancient tissues that may occasionally survive as near-complete skeletons, but are more often highly fragmentary, leaving the analyst unable to identify the original species based on morphology alone [24]. In the three types of examples given above, the primary differences are the degrees of morphological integrity remaining from the source animal tissues, proportional to the level of molecular analysis required.

## 2. Molecular Techniques in Species Identification

With the development of the Polymerase Chain Reaction technique of amplifying DNA in the 1980s, DNA became a viable means of species identification that continues to be widely employed due to the ubiquity of the molecule (*i.e.*, in all tissue types) and level of genetic information recovered. However, when tissues are processed under particular conditions, such as thermal processing or high pressure, both of which being used in the example of meat and bone meal (MBM) production, these approaches become less reliable [21]. The main alternative biomolecules that have been widely investigated as a source of species information are proteins, which are coded for by DNA and therefore contain less albeit some level of genetic information useful for species determination. Early protein-based methods were based on immunological techniques [25,26], which continue to be developed and utilised by some [27,28], but the increasing use of protein sequence-based methods, particularly following the development of soft-ionization mass spectrometry in the late 1980s and technical improvements in the 1990s resulting in the emerging field of proteomics, has been so far more widely utilised in the fields of bioarchaeology [29,30] and microbial studies [31,32] than in the animal feed industry (although note [33]).

The primary aims of this research were to compare the two most commonly used mass spectrometric techniques of species identification of collagenous tissues, peptide mass fingerprinting (PMF) and peptide sequencing by in-depth liquid chromatography tandem mass spectrometry (LC-MS/MS). The former methodology, in which an isolated protein or protein mixture is enzymatically digested into peptides that are measured directly using soft-ionization mass spectrometry, has existed for over two decades [34] and remains to be considered as the fastest and cheapest method of protein identification [35]; these typically involve analysis by Matrix Assisted Laser Desorption Ionization (MALDI) mass spectrometers. The latter methods (LC-MS/MS), particularly “shotgun proteomics”, using coupled HPLC instrumentation to separate complex peptide mixtures prior to mass spectrometric analysis [36], resulting in much larger generated datasets but that often contain large amounts of unused information [37]. In this study, bone collagen from cattle (*Bos taurus*) and sheep (*Ovis aries*) were compared as closely related ruminants that both have complete collagen α1(I) and α2(I) sequences publicly available. Along with pigs (*Sus scrofa*), also included in this study, these compose the primary species of interest to the animal feed industry.

## 3. Results

### 3.1. Collagen Variation between Artiodactyls

**Figure 1:**
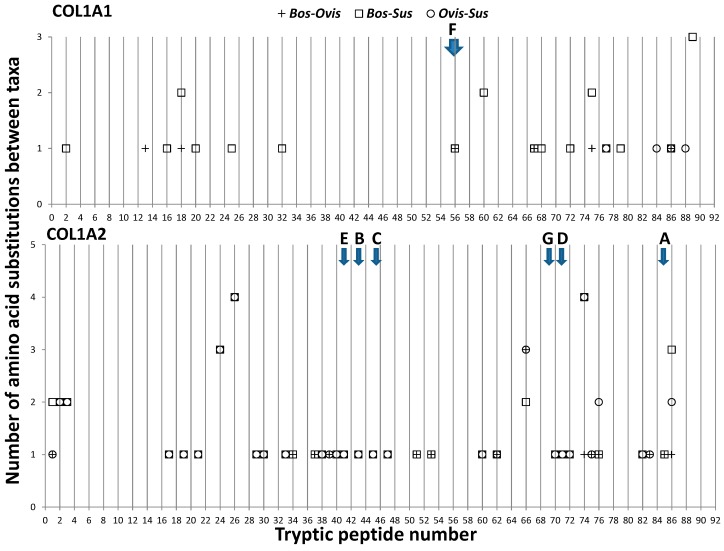
Plots of the number of amino acid substitutions between cattle (*Bos*), sheep (*Ovis*) and pig (*Sus*) collagen tryptic peptides for the α1(I) (**top**) and α2(I) (**bottom**) chains. Species biomarkers A–G presented in Buckley *et al.* [29] are indicated with arrows and labelled accordingly.

Sequence comparison of α1(I) and α2(I) chains from cattle, sheep and pig type 1 collagen confirms that the α2(I) is typically much more variable than the α1(I) chain (Figure 1), but more noticeably that the previously described collagen peptide biomarkers most frequently observed in the peptide mass fingerprints [29] are typically not the most variable tryptic peptides potentially present. Comparison with functional attributes [38] did not readily indicate a strong relationship with amino acid variation.

### 3.2. Peptide Mass Fingerprinting

**Figure 2:**
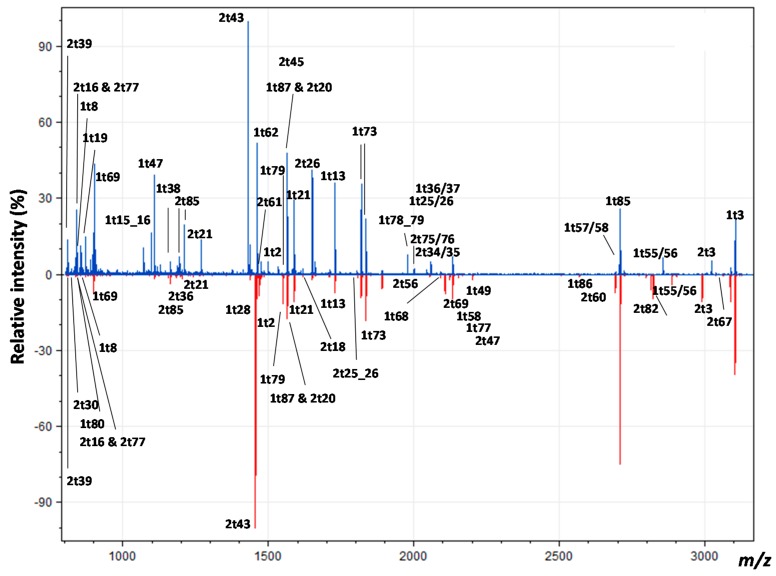
Matrix Assisted Laser Desorption Ionization Time of Flight (MALDI-ToF) mass spectra of collagen tryptic digests from *Bos* (**top**) and *Sus* (**bottom**) bone, annotated with peptide labels relating to their position in the α chains. 2t3 is noted as being subject to an additional mass shift due to the change of a proline residue that is predominantly hydroxylated in *Bos* (“/” indicates missed cleavage site, *i.e.*, the presence of an internal K or R residue; “&” indicates that more than one peptide are observed with a similar *m*/*z* value).

**Figure 3:**
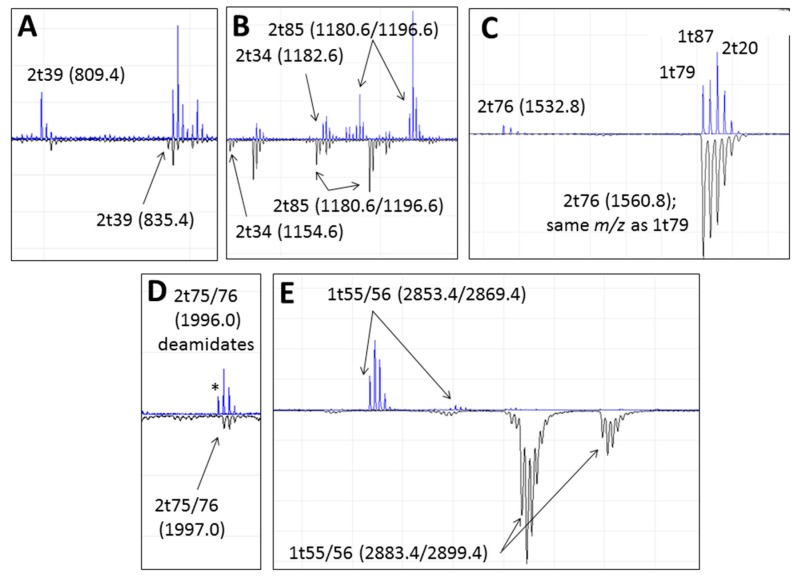
Sections of the MALDI fingerprints that highlight homologous markers between *Bos* (**top**) and *Ovis* (**bottom**) tryptic collagen peptides showing: (**A**) 2t39; (**B**) 2t34 and 2t85; (**C**) 2t76 (noting that the *Ovis* marker is at the same *m*/*z* as other collagen peptides); (**D**) 2t75/76 (* note that the *Bos* form of 2t75, HGNR, includes an amino acid susceptible to deamidation that could be mistaken as the homologous marker in *Ovis* and *Sus*); and (**E**) 1t55/56 (“/” indicates missed cleavage site, *i.e.*, the presence of an internal K or R residue).

Although the MALDI fingerprints typically yield varying numbers of peaks per species, likely due to the different pre-treatment methods rather than species-specific differences, they typically yield ~80–200 peaks ([29,30]; e.g., Figure 2). However, as many of these are post-translational modification (PTM) variants of fewer peptides, the observed peptide numbers are typically reduced to ~50 (including longer peptides that are due to missed tryptic cleavages). Regarding the 19 potential peptides that could separate cattle from sheep, only six were observed in the fingerprints (Figure 3; 2t34, 2t39, 2t55/56, 2t75, 2t76 observed as the missed cleaved peptide 2t75/76 and 2t85) whereas of the 16 peptides that include two or more amino acid variations between one of the bovids (cattle and sheep; Figure 1) and suids (pigs), only four were observed in the fingerprints (Figure 2; 1t16, 2t3, 2t26 and 2t76 where the number preceding the “t” reflects the alpha chain, with the “t” being an abbreviation of the enzyme trypsin, and the number following the “t” is the consecutive peptide number assuming cleavage at K and/or R residues). Surprisingly, only one (1t86) of the four unique α1(I) peptide sequences (of the three taxa within this study; 1t18, 1t67, 1t75 and 1t86) and none of the six unique α2(I) peptide sequences (2t1, 2t51, 2t62, 2t66, 2t74 and 2t86) were observed in the fingerprints.

It is noteworthy that in some cases an amino acid change can result in a peptide marker becoming similar in *m*/*z* value to other peptides present (Figure 3C). In the case of 2t76, increasing from *m*/*z* 1532.8 to *m*/*z* 1560.8 from *Bos* to *Ovis*, this shares the same *m*/*z* as 1t79, within the isotopic envelope of which is both 1t87 and 2t20. Therefore in a situation such as this, where the absence of the evident marker is not ideal, it is possible to note the difference in the monoisotopic peak clusters, in which that at *m*/*z* 1560.8 is relatively more abundant in *Ovis* than the *m*/*z* 1561.8 and 1562.8 peaks.

### 3.3. Peptide Sequencing

The peptide sequencing results are typically much more complex than the above fingerprints, due to the large amount of data (e.g., Tables S1–S3) that needs to be scrutinised in relation to problems associated with probability-matching peptides with highly repetitive sequences (despite the ability of the software to account for such “variable modifications” on pre-defined residue types; these could be undefined residue types with “Error Tolerant” type searches in Mascot). For example, the regular presence of hydroxylation modifications on the abundant proline and lysine residues can result in the incorrect assignment, even with a relatively high score, of peptide sequences of similar mass (e.g., where a nearby residue undergoes an alanine to serine transition between taxonomic groups). However, as expected, a much greater number of peptides were observed than with the fingerprints. Only 11 of the 92 α1(I) peptides and six of the 87 α2(I) peptides were not observed, but none of the former and only one of the latter showed amino acid variation between the three artiodactyls in this study (Table 1 and Table 2; Tables S1–S3); nine of the ten unique peptide sequences were repeatedly observed in the LC-based approaches (Table 3).

**Figure 4:**
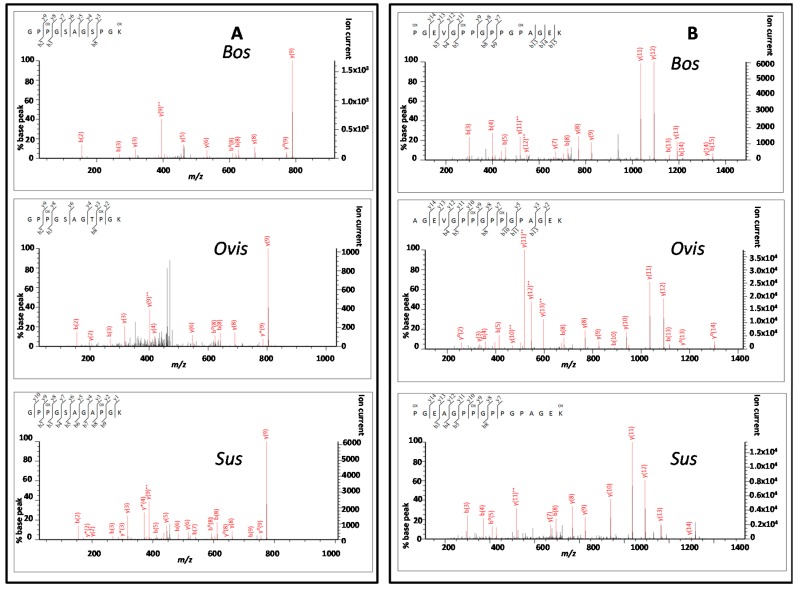
Tandem mass spectra of the two highest scoring α1(I) chain peptides that are unique to each taxa within this study showing peptides: (**A**) 1t67; and (**B**) 1t86.

**Figure 5:**
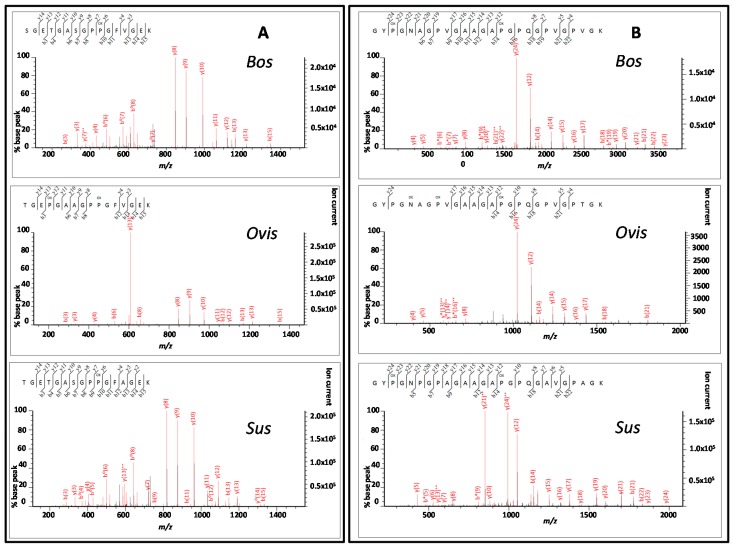
Tandem mass spectra of the two highest scoring α2(I) chain peptides that are unique to each taxa within this study showing peptides: (**A**) 2t66; and (**B**) 2t74.

Sequence coverages were 70%, 92% and 93% for the collagen α1(I) chain (COL1A1) from *Bos*, *Ovis* and *Sus* samples, respectively, and 93%, 95% and 98% for the COL1A2 sequences. When a peptide ion score filter set at the threshold for identity was used (40 for each analysis), these were reduced to 52%, 79% and 85% for COL1A1 and 68%, 67% and 80% for COL1A2, respectively. Of the nine unique peptides between these three taxa observed in all three samples, the two α1(I) peptides of best quality were 1t67 and 1t86 (Figure 4) and for the α2(I) peptides these were 2t66 and 2t74 (Figure 5).

## 4. Discussion

### 4.1. Regions of Collagen Sequence Variation

Molecular sequence variation in proteins is likely to be highly conserved in relation to their functional properties, yet the functions of type 1 collagen are widely diverse and not fully understood. Collagen is a large ubiquitous protein that has been in existence for over half a billion years evolving into many different forms throughout the animal kingdom with fibrillary collagen even known from choanoflagellates [39], the closest living relatives of animals. During this time, it has evolved into a protein that facilitates numerous interactions with proteoglycans and mucopolysaccharides [40], whereby the amino acid sequence can be investigated to infer functional attributes [38]. It is clear that throughout its evolution, type 1 collagen has acquired an increasing number of functional relationships with other biomolecules that in this case make it difficult to associate regions of sequence variation with particular functional constraints beyond those of the structural Gly–Xaa–Yaa motif where, at least in the α1(I) chain, Xaa is frequently proline and Yaa hydroxyproline.

Considering some of the most variable peptides within the α1(I) chain, peptide sequence 1t18 is close to multiple protein interaction sites (decorin, osteonectin (secreted protein acidic rich in cysteine (SPARC), heat shock protein 47 (HSP47), and α2β1 integrin), peptide sequence 1t60 is close to a binding site for SPARC and HSP47, peptide sequence 1t75 is near a binding site for HSP47 and dermatan sulfate proteoglycan (DSPG), peptide sequence 1t79 is within the thermally labile domain and close to a HSP47 site (and DSPG), and peptide sequence 1t86 is near a decorin-binding site and just after a HSP47 site. Note that the α1(I) marker (F) reported in our previous publication [29] derives from a cartilage oligomeric matrix protein (COMP)-binding site. Within the α2(I) chain, peptide sequence 2t19 is nearby a HSP47-, SPARC- and decorin-binding region whereas the peptide sequences 2t24–40 are all near a HSP47-binding site. The peptide sequence for 2t41 has a glycation site on the internal and preceding lysine residues and follows a HSP47-binding site. Peptide sequences 2t45–53 span integrin-binding and phosphoprotein-binding sites whereas peptide sequences 2t60–73 have associations with SPARC, COMP, and another phosphoprotein-binding region, also spanning an integrin-binding zone. Towards the carboxy-terminal end of the chain, there is greater sequence variation at peptides 2t74–76, which starts near the end of a DSPG-binding site and near the beginning of the thermally-labile domain. Interestingly, most of the α2(I) species peptide biomarkers from previous publications (e.g., [29]) either span an integrin-binding site (D and G) or a keratan sulphate proteoglycan-binding region (B, C and E). However, these reported binding sites are frequently observed throughout the protein, where many of the highly conserved peptide sequences would also be associated with them; as such they do not prove useful in the identification of intra-chain function properties directly influencing sequence variability. However, one clear observable difference is that, as shown in Figure 1, the α2(I) chain sequence variation is much greater than that of the α1(I) chain. Given that collagen α1(I) homotrimers are known to be more stable than the natural heterotrimer and that the unwinding of the triple helix is necessary for placing the individual chains inside the catalytic cleft of the enzyme [41], perhaps this greater sequence variation is due to a potential role as the chain that is preferentially unzipped by mammalian collagenase. The greater variation towards the thermally-labile region could speculatively [42] also relate to this purpose although note that there could be other evolutionary constraints driving this [43].

### 4.2. Comparing Peptide Fingerprinting with Sequencing

Although both methods are easily capable of separating the limited number of domesticate taxa in this study, there are clear advantages and disadvantages of utilising either PMF by MALDI, or in-depth “sequencing” by LC-based methods. The PMF approach offers the advantage that it is a relatively simple approach that is amenable to high-throughput applications at low cost, but with the disadvantage that some of the most variable peptide markers are not regularly observed. This could be particularly problematic for samples of mixed-species origin such as rendered MBM. The LC-based methods have the advantage that they do result in matches to almost all of the most useful species-specific biomarkers, but the disadvantage that these are probability-based matches which could result from false positive matches to similar peptides from potentially different species. For example, tandem spectra searches using algorithms such as implemented by Sequent or Mascot aim to report the probability that the match is random or not. However, the detection of potential species biomarkers will be confounded by amino acid substitutions between taxa that result in similar masses, whereby the closer the variations are within the sequence, the more of the fragment ion series that are likely to match and result in a higher peptide ion score. For example, the peptide GSTGEIGPAGPPGPPGLR (2t26) in ruminants, particularly when deamidated, would have the same precursor, and could generate a false positive by similarity to GPNGEVGSAGPPGPPGLR in pigs despite having four amino acid substitutions between the two peptide sequences (that all occur within the first eight residues). This is particularly complicated with collagen due to the high number of fixed as well as variable hydroxylation modifications. One particular example of this is with the two peptides GAPGPDGNNGAQGPPGLQGVQGGK (2t40) in cattle and sheep and GAPGPDGNNGAQGPPGPQGVQGGK in the pig sequence (the leucine to hydroxyproline substitution, equally problematic with isoleucine). More common examples are those relating to the substitutions leading to a change between alanine and serine when there is a neighbouring proline that may be a site for variable hydroxylation. This scenario would mean that the absence of a specific b- or y-ion (sequence fragment ions possessing its charge on either the amino- or carboxy-terminus respectively, following [44]) can remove the possibility of identification, yet may still yield a high probability match score. Examples in this study include the peptide GAPGPAGPK (1t32) in ruminants (cattle and sheep) which is GSPGPAGPK in pigs, GAPGADGPAGAPGTPGPQGIAGQR (1t68) in ruminants as GSPGADGPAGAPGTPGPQGIAGQR in pigs, DGSPGAK (1t75) specific to cattle as DGAPGAK in sheep, GPPGSAGSPGK (1t86) in cattle as GPPGSAGAPGK in pigs, TGPPGPSGISGPPGPPGPAGK (2t62) in cattle as TGPPGPAGISGPPGPPGPAGK in sheep, GENGPVGPTGPVGAAGPSGPNGPPGPAGSR (2t60) in ruminants as GENGPVGPTGPVGAAGPAGPNGPPGPAGSR in pigs. The alanine-serine switch masked by the presence of variable hydroxylation cannot be readily distinguished on precursor mass alone because they both involve the presence of a single oxygen atom, and has been noted as potentially causing issues with species discrimination using tandem mass spectra [45]. The distinction between a hydroxyproline and leucine/isoleucine residue is 0.036, which could readily be separated depending on the resolution of the instrumentation used, but this is a much less frequent issue.

### 4.3. Variable Hydroxylation

**Figure 6:**
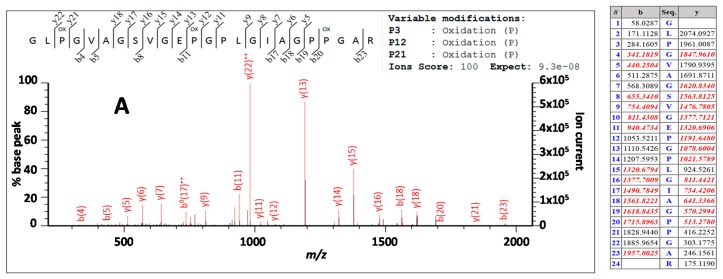

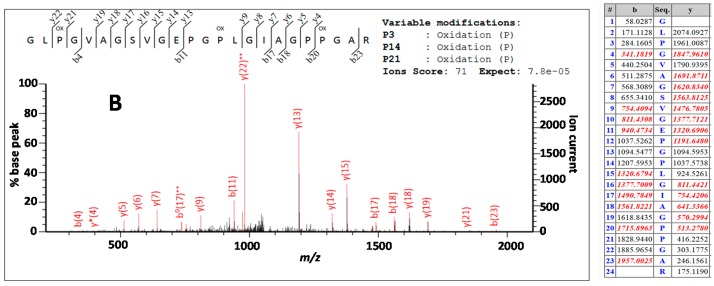
Example tandem mass spectra taken from Mascot output showing matches (numbers coloured red) to the same collagen peptide (2t69) but with (**A**) a variable hydroxylation matched on the 12th residue, compared with (**B**) a variable hydroxylation on the 14th residue (the peptide significance score for this (*Bos*) search was 40 and the highest false positive ion score as 31; the false discovery rate above identity threshold was 2.27%).

As noted above, hydroxylation modifications of proline residues, and to a lesser extent lysine residues, is a common observation in proteomics datasets that result from the analysis of extracellular tissues. These have been studied for types I and III [46], type IV [47], type V [46,48] as well as a range of non-collagenous proteins such as osteocalcin [49,50]. However, exhaustive maps that attempt to span the entirety of type 1 remain elusive due to issues with reproducibility within such proteomics methods. For such reasons this manuscript does not attempt to do so here, but aims to consider the approaches of combining fingerprinting with LC-based methods to investigate heterogeneity between closely related species. The LC-based analyses of the three specimens presented herein contain almost two thousand fragment ion matches to collagen each (*Bos*: 1861, *Ovis*: 1986, *Sus*: 2055), whereby determining which of these peptide ions are reliable interpretations for estimates of relative abundance is fraught with issues. The fingerprinting is less influenced by these issues, but is less able to resolve the location of the modifications within each peptide. For example, peptide 2t85 (IGQPGAVGPAGIR) is present in the PMF in both its unmodified form as well as a form in which the fourth residue (underlined) is hydroxylated. In the case of this example (2t85) given in Figure 3B, the hydroxylated form is present at approximately 2–3 times the abundance of the unmodified form, but note the complexity in assessing this due to the presence of a deamidating residue, in which case the relative abundance under the whole isotopic envelope is preferential rather than under the monoisotopic peak alone; this would be more problematic with LC-based methods that would resolve these as distinct analytes during the separation phase. A second example from the PMFs (see [29]) is that of 2t69 (GLPGVAGSVGE**P**G**P**LGIAGPPGAR) which appears to have at least four hydroxylation sites (underlined), with the 3 OH form being much more intense than the 2 OH (positions 3 and 21 within the peptide) and 4 OH forms; when studying variation between species it becomes more clear that despite having high Mascot scores (*Bos*: 71; *Ovis*: 76; *Sus*: 92) these modifications can be readily misplaced. In this example they may be inferred to suggest that both variants are present (*i.e.*, with one hydroxylation at either underlined bold P) but manual interpretation of the tandem data (e.g., Figure 6) only shows the y13 ion (~1191) consistent with the modification (Figure 6A), with no clear observation of the y11 ion (at *m*/*z* 1037 rather than ~1021) expected for the alternative modification site (Figure 6B; noting that even if present at low abundance could be due to the downstream hydroxylation site); the b ion series not being useful at discrimination in this case.

### 4.4. Alternative Approaches

Some of the most advanced approaches currently used in proteomics that are ideal for the quantitative determination of known species-specific biomarkers are the targeted methods of selected/multiple reaction monitoring (SRM/MRM) that complement the untargeted methods so far described [51]. In SRM/MRM, one ion is selected for following one stage of mass analysis, fragmented in a second, and one or more of the fragment ions from the precursor screened for. As long as appropriate fragment ions that are specific to the desired peptide can be readily identified as being unique (e.g., Figure 4 and Figure 5), the method should be ideal for species discrimination even in mixed tissues, but with the considerable issues that hydroxylation modifications could also bring to such analyses that would need to be taken into account. In addition to standard data-dependent and SRM/MRM analyses, further developments are on-going in the area of data-independent analyses (where the previously described ‘shotgun proteomics’ methods used in this study were based on data-dependent analysis for the determination of selected peptides for fragmentation) and hyper reaction monitoring that result in higher sequence coverage and selectivity, respectively [52]. These will ultimately increase the extent to which proteomics could be used in species determination of animal tissues and animal proteins, but an understanding of the complexity of such investigations specific to the collagen as highlighted above will remain crucial.

## 5. Materials and Methods

Powder from the three species was drilled from bone samples of each and demineralised with 0.6 M hydrochloric acid (HCl) for 18 h and then centrifuged at 14,000 rpm. Collagen peptide mass fingerprinting was carried out following a modified method of Buckley *et al.* [29], whereby following removal of the acid-soluble fraction, the insoluble residue was heated at 65 °C for 3 h in 50 mM ammonium bicarbonate. The solubilised gelatine was then centrifuged as before, separated into a fresh Eppendorf tube, and digested with 2 µL of 0.4 µg/µL trypsin for a further 18 h at 37 °C. The digests were stopped with the addition of 1% trifluoroacetic acid (TFA) to a final concentration of 0.1% TFA, purified using C18 solid phase extraction cartridges with 50% acetonitrile (ACN in 0.1% TFA), evaporated and resuspended with 20 µL 0.1% TFA. 1 µL co-crystallised on a stainless steel MALDI target plate with a further 1 µL α-cyano hydroxycinnamic acid matrix. MALDI analysis was carried out using a Bruker Ultraflex II instrument (Bruker Daltonik, Bremen, Germany).

In-depth peptide sequencing analysis was carried out following the methods of Wadsworth and Buckley [53]. LC-MS/MS was carried out on a Waters nanoAcquity UPLC (Manchester, UK) coupled to a Thermo Scientific Orbitrap Elite mass spectrometer (Hemel Hempstead, UK) on which the peptides were concentrated using a pre-column (20 mm × 180 µm) then separated on a 1.7 µM Waters nanoAcquity BEH (Ethylene Bridged Hybrid) C18 analytical column (75 mm × 250 µm), using a gradient from 99% buffer A (0.1% formic acid (FA) in H_2_O)/1% buffer B (0.1% FA in ACN) to 25% buffer B in 45 min at 200 nL·min^−1^. Peptides were selected for fragmentation automatically by data dependent analysis. Proteomics data files were searched using Mascot v2.5.1 (Matrix Science, London, UK) against a local database that contained collagen sequences for the three species of interest, cropped to the ends of each telopeptide, in addition to SwissProt (which also contains cattle (*Bos taurus*) sequences). The COL1A1 and COL1A2 *Bos* sequences were taken from UniProt accession numbers P02453 and P02465, *Ovis* sequences from WSP481 and W5NTT7 and the *Sus* COL1A2 sequence from F1SFA7. The *Sus* COL1A1 sequence was obtained through BLAT (UCSC genome browser) search of the *Bos* sequence, and its gaps filled through further protein Basic Local Alignment Search Tool (BLAST) searches, both against pig sequences only. The *Ovis* sequences were also completed using BLAST searches. Standard searches were carried out using two missed cleavages, error tolerances of 5 ppm and 0.5 *m*/*z* units (MS and MS/MS respectively) and variable oxidation of methionine, hydroxylation of proline and lysine and deamidation of asparagine and glutamine modifications.

## 6. Conclusions

In conclusion, even though the currently used set of collagen PMF markers are likely to need expanding upon, the PMF approach makes for the ideal technique to be used to obtain species-level identifications in initial investigations. This is particularly due to its amenability to high-throughput processing [54] and resultant low cost of analysis per sample. Subsequent analyses using LC-based approaches may be utilised if it is determined that greater taxonomic resolution is required. These will likely require much greater input relating to either sequence database improvements or methodological design in the case of targeted approaches, but the nine new unique peptide markers described here may prove a valuable target for such future studies supported by those identified previously.

## Figures and Tables

**Table 1:** Collagen alpha 1(I) (COL1A1) peptide sequences showing amino acid variations between artiodactyl taxa (hyphen indicates identical amino acid residue as the main sequence = *Bos*; sequences followed by O = *Ovis* and S = *Sus*). (√) indicates observation of precursor in (at least 2 of 3) fingerprints (peptide mass fingerprinting (PMF) data); shaded cells indicate lack of observation in (at least 2 of 3) liquid chromatography (LC)-based results (see Tables S1–S3); single lettering under “Peptide label” indicates PMF species biomarker from Buckley *et al.* [29].

Peptide Label *	Sequence	Peptide Label *	Sequence
1t1	QLSYGYDEK	1t47 (√)	GVQGPPGPAGPR
1t2 (√)	STGISVPGPMGPSGPR; -A--------------(S)	1t48	GANGAPGNDGAK
1t3 (√)	GLPGPPGAPGPQGFQGPPGEPGEPGASGPMGPR	1t49 (√)	GDAGAPGAPGSQGAPGLQGMPGER
1t4	GPPGPPGK	1t50	GAAGLPGPK
1t5	NGDDGEAGK	1t51	GDR
1t6	PGR	1t52	GDAGPK
1t7	PGER	1t53	GADGAPGK
1t8 (√)	GPPGPQGAR	1t54	DGVR
1t9	GLPGTAGLPGMK	1t55 (F) (√)	GLTGPIGPPGPAGAPGDK
1t10	GHR	1t56 (F) (√)	GEAGPSGPAGPTGAR; --T------------(O; S)
1t11	GFSGLDGAK	1t57 (√)	GAPGDR
1t12	GDAGPAGPK	1t58 (√)	GEPGPPGPAGFAGPPGADGQPGAK
1t13 (√)	GEPGSPGENGAPGQMGPR; ----------T-------(O)	1t59	GEPGDAGAK
1t14	GLPGER	1t60	GDAGPPGPAGPAGPPGPIGNVGAPGPK; -----------T-------S-------(S)
1t15 (√)	GR	1t61	GAR
1t16 (√)	PGAPGPAGAR; --P-------(S)	1t62 (√)	GSAGPPGATGFPGAAGR
1t17	GNDGATGAAGPPGPTGPAGPPGFPGAVGAK	1t63	VGPPGPSGNAGPPGPPGPAGK
1t18	GEGGPQGPR; --A------(O); --A----A-(S)	1t64	EGSK
1t19 (√)	GSEGPQGVR	1t65	GPR
1t20	GEPGPPGPAGAAGPAGNPGADGQPGAK; -------------------------G-(S)	1t66	GETGPAGR
1t21 (√)	GANGAPGIAGAPGFPGAR	1t67	PGEVGPPGPPGPAGEK; A---------------(O); ---A------------(S)
1t22	GPSGPQGPSGPPGPK	1t68 (√)	GAPGADGPAGAPGTPGPQGIAGQR; -S----------------------(S)
1t23	GNSGEPGAPGSK	1t69 (√)	GVVGLPGQR
1t24	GDTGAK	1t70	GER
1t25 (√)	GEPGPTGIQGPPGPAGEEGK; -------V------------(S)	1t71	GFPGLPGPSGEPGK
1t26 (√)	R	1t72	QGPSGASGER; -----P---- (S)
1t27	GAR	1t73 (√)	GPPGPMGPPGLAGPPGESGR
1t28 (√)	GEPGPAGLPGPPGER	1t74	EGAPGAEGSPGR
1t29	GGPGSR	1t75	DGSPGAK; --A----(O); --A—-P-(S)
1t30	GFPGADGVAGPK	1t76	GDR
1t31	GPAGER	1t77 (√)	GETGPAGPPGAPGAPGAPGPVGPAGK; --S-----------------------(S)
1t32	GAPGPAGPK; -S-------(S)	1t78 (√)	SGDR
1t33	GSPGEAGR	1t79 (√)	GETGPAGPAGPIGPVGAR; -----------V------(S)
1t34	PGEAGLPGAK	1t80 (√)	GPAGPQGPR
1t35	GLTGSPGSPGPDGK	1t81	GDK
1t36 (√)	TGPPGPAGQDGR	1t82	GETGEQGDR
1t37 (√)	PGPPGPPGAR	1t83	GIK
1t38 (√)	GQAGVMGFPGPK	1t84	GHR
1t39	GAAGEPGK	1t85 (√)	GFSGLQGPPGPPGSPGEQGPSGASGPAGPR
1t40	AGER	1t86 (√)	GPPGSAGSPGK; -------T---(O); -------A---(S)
1t41	GVPGPPGAVGPAGK	1t87 (√)	DGLNGLPGPIGPPGPR
1t42	DGEAGAQGPPGPAGPAGER	1t88	GR
1t43	GEQGPAGSPGFQGLPGPAGPPGEAGK	1t89	TGDAGPAGPPGPPGPPGPPGPPSGGYDLSFLPQPPQEK------V------------------F-F----------(S)
1t44	PGEQGVPGDLGAPGPSGAR	1t90	AHDGGR
1t45	GER	1t91	YYR
1t46	GFPGER	1t92	A

**Table 2:** COL1A2 peptide sequences showing amino acid variations between artiodactyl taxa (hyphen indicates identical amino acid residue as the main sequence = *Bos*; sequences followed by O = *Ovis* and S = *Sus*). (√) indicates observation of precursor in (at least 2 of 3) fingerprints (PMF data); shaded cells indicate lack of observation in (at least 2 of 3) LC-based results (see Tables S1–S3); single lettering under “Peptide label” indicates PMF species biomarker from Buckley *et al.* [29].

Peptide Label	Sequence	Peptide Label	Sequence
2t1	QFDAK; ---G-(O); -Y-G-(S)	2t45 (C) (√)	GPPGESGAAGPTGPIGSR; -----------A------(S)
2t2	G-G-GPGPMGLMGPR; -V-A-----------(S)	2t46	GPSGPPGPDGNK
2t3 (√)	GPPGASGAPGPQGFQGPPGEPGEPGQTGPAGAR; -----V-----------A---------------(S)	2t47 (√)	GEPGVVGAPGTAGPSGPSGLPGER-----L------------------(S)
2t4	GPPGPPGK	2t48	GAAGIPGGK
2t5	AGEDGHPGK	2t49	GEK
2t6	PGR	2t50	GETGLR
2t7	PGER	2t51	GDIGSPGR; --V-----(O); --V-----(S)
2t8	GVVGPQGAR	2t52	DGAR
2t9	GFPGTPGLPGFK	2t53	GAPGAIGAPGPAGANGDR; -----V------------(O; S)
2t10	GIR	2t54	GEAGPAGPAGPAGPR
2t11	GHNGLDGLK	2t55	GSPGER
2t12	GQPGAPGVK	2t56 (√)	GEVGPAGPNGFAGPAGAAGQPGAK
2t13	GEPGAPGENGTPGQTGAR	2t57	GER
2t14	GLPGER	2t58	GTK
2t15	GR	2t59	GPK
2t16	VGAPGPAGAR	2t60 (√)	GENGPVGPTGPVGAAGPSGPNGPPGPAGSR-----------------A------------(S)
2t17	GSDGSVGPVGPAGPIGSAGPPGFPGAPGPK; -N----------------------------(S)	2t61 (√)	GDGGPPGATGFPGAAGR
2t18 (√)	GELGPVGNPGPAGPAGPR	2t62	TGPPGPSGISGPPGPPGPAGK; ------A--------------(O); I--------------------(S)
2t19	GEVGLPGLSGPVGPPGNPGANGLPGAK-------V-------------------(S)	2t63	EGLR
2t20 (√)	GAAGLPGVAGAPGLPGPR	2t64	GPR
2t21 (√)	GIPGPVGAAGATGAR; -----A---------(S)	2t65	GDQGPVGR
2t22	GLVGEPGPAGSK	2t66	SGETGASGPPGFVGEK; T--P--A---------(O); T-----------A---(S)
2t23	GESGNK	2t67 (G) (√)	GPSGEPGTAGPPGTPGPQGLLGAPGFLGLPGSR
2t24	GEPGAVGQPGPPGPSGEEGK; -----A-PQ-----------(S)	2t68	GER
2t25 (√)	R	2t69 (D) (√)	GLPGVAGSVGEPGPLGIAGPPGAR
2t26 (√)	GSTGEIGPAGPPGPPGLR; -PN--V--S----------(S)	2t70	GPPGNVGNPGVNGAPGEAGR; ----A---------------(S)
2t27	GNPGSR	2t71	DGNPGNDGPPGR; -----S------(S)
2t28	GLPGADGR	2t72	DGQPGHK; ---A---(S)
2t29	AGVMGPAGSR; ------P---(S)	2t73	GER
2t30 (√)	GATGPAGVR; -P-------(S)	2t74	GYPGNAGPVGAAGAPGPQGPVGPVGK; -----------------------T--(O); -----P—A-----------A---A--(S)
2t31	GPNGDSGR	2t75 (√)	HGNR; --S-(O)
2t32	PGEPGLMGPR	2t76 (√)	GEPGPAGAVGPAGAVGPR; -----V------------(O); -------S----------(S)
2t33	GFPGSPGNIGPAGK	2t77	GPSGPQGIR
2t34 (√)	EGPVGLPGIDGR; ---A--------(O;S)	2t78	GDK
2t35 (√)	PGPIGPAGAR	2t79	GEPGDK
2t36 (√)	GEPGNIGFPGPK	2t80	GPR
2t37	GPSGDPGK; --T-----(O;S)	2t81	GLPGLK
2t38	AGEK; N---(S)	2t82 (√)	GHNGLQGLPGLAGHHGDQGAPGAVGPAGPR; ----------------------P-------(S)
2t39 (√)	GHAGLAGAR; -------P-(O)	2t83	GPAGPSGPAGK; -----T-----(O)
2t40	GAPGPDGNNGAQGPPGLQGVQGGK; ----------------P-------(S)	2t84	DGR
2t41 (E) (√)	GEQGPAGPPGFQGLPGPAGTAGEAGK; -----------------------V--(S)	2t85 (A) (√)	IGQPGAVGPAGIR; T------------(O; S)
2t42 (E) (√)	PGER	2t86	GSQGSQGPAGPPGPPGPPGPPGPSGGGYEFGFDGDFYR; ----------------------------D---------(O)----------------------------D--YE-----(S)
2t43 (B) (√)	GLPGEFGLPGPAGAR; -------------P-(S)		
2t44	GER	2t87	A

**Table 3:** Mascot ion scores for the nine unique peptides observed in all taxa (m.c. indicates only observed through missed cleavage site to the exception of * also included for similarity to homologous peptides).

Peptide Label	*Bos*	*Ovis*	*Sus*
1t18	41 (m.c.)	35	47
1t67	45	88	48
1t75	46 (m.c.)	55 (m.c.)	5/14 (m.c.) *
1t86	56	38	62
2t1	43 (m.c.)	56 (m.c.)	69 (m.c.)
2t51	35 (m.c.)	25	35
2t62	50	37	56
2t66	81	59	80
2t74	80	48	77

## Supplementary Materials

Supplementary materials can be found at http://www.mdpi.com/1422-0067/17/4/445/s1.
